# Plasma extracellular vesicle test sample to standardize flow cytometry measurements

**DOI:** 10.1016/j.rpth.2023.100181

**Published:** 2023-05-18

**Authors:** Britta Bettin, Edwin van der Pol, Rienk Nieuwland

**Affiliations:** 1 Laboratory of Experimental Clinical Chemistry, Amsterdam UMC location University of Amsterdam, Amsterdam, The Netherlands; 2 Biomedical Engineering and Physics, Amsterdam UMC location University of Amsterdam, Amsterdam, The Netherlands; 3 Amsterdam Vesicle Center, Amsterdam UMC location University of Amsterdam, Amsterdam, The Netherlands

**Keywords:** blood plasma, calibration, extracellular vesicles, flow cytometry, quality control, standardization

## Abstract

**Background:**

Extracellular vesicles (EVs) in body fluids are explored as disease biomarkers, but EV concentrations measured by flow cytometers (FCMs) are incomparable.

**Objectives:**

To improve data comparability, new reference materials with physical properties resembling EVs and reference procedures are being developed. The validation of new reference materials and procedures requires biological test samples. We developed a human plasma EV test sample (PEVTES) that i) resembles subcellular particles in plasma, ii) is ready-to-use, iii) is flow cytometry–compatible, and iv) is stable.

**Methods:**

The PEVTES was prepared from human plasma of 3 fasting donors. EVs were immunofluorescently stained with antibodies against platelet-specific (CD61) and erythrocyte-specific (CD235a) antigens or lactadherin. To reduce the concentration of soluble proteins, lipoproteins, and unbound reagents, stained EVs were isolated from plasma by size-exclusion chromatography. After isolation, the PEVTES was filtered to remove remnant platelets. PEVTESs were diluted in cryopreservation agents, dimethyl sulfoxide, glycerol, or trehalose and stored at −80 °C for 12 months. After thawing, stained EV concentrations were measured with a calibrated FCM (Apogee A60-Micro).

**Results:**

We demonstrate that the developed PEVTES resembles subcellular particles in human plasma when measured using FCM and that the concentrations of prestained platelet-derived, erythrocyte-derived, and lactadherin^+^ EVs in the PEVTES are stable during storage at −80 °C for 12 months when stored in trehalose.

**Conclusion:**

The PEVTES i) resembles subcellular particles in plasma, ii) is ready-to-use, iii) is flow cytometry–compatible, and iv) is stable. Therefore, the developed PEVTES is an ideal candidate to validate newly developed reference materials and procedures.

## Essentials

• Extracellular vesicle (EV) concentration measurements by flow cytometry require calibration.
• Calibration requires reference materials and test samples.
• We developed a stable, ready-to-use EV test sample from human plasma.
• The plasma EV test sample is an ideal candidate to validate reference materials and procedures.

## 1 Introduction

The term “extracellular vesicles” (EVs) is an umbrella term for naturally released cell-derived particles with a phospholipid bilayer. EVs are present in body fluids, such as blood and urine [1,2]. Properties of EVs, such as the cellular origin, concentration, composition, and function, are disease-dependent. Therefore, EVs are being explored as biomarkers for diseases, including cancer and cardiovascular disease [1,3,4]. However, to explore EVs as biomarkers, reliable and reproducible measurements of EVs are needed. At present, a major hurdle in EV biomarker research is that measurement results are incomparable between instruments and institutes, which hampers setting up multicenter studies. Multicenter studies are a prerequisite for clinical biomarker studies.

At present, most clinical laboratories use flow cytometry to measure the concentration of immunofluorescently stained EVs in body fluids because flow cytometry is capable of detecting and characterizing particles at a throughput of thousands/s [[5], [6], [7]]. A flow cytometer (FCM) detects fluorescence and light scattering signals of single particles, such as EVs, when these signals exceed the lower detection limit of the detectors. There are at least 4 major reasons why EV concentration measurement results are incomparable. First, compared with cells, stained EVs emit little fluorescence and scatter light inefficiently, which makes EVs hard to detect [8,9]. Second, fluorescence and light scattering signals of a part of the total EV population are below the detection limit of commercially available FCMs. Third, signals measured by FCM have arbitrary units, which hampers the quantification and comparison of detection ranges between FCMs [10,11]. Fourth, most EV samples contain non-EV particles, such as lipoproteins in plasma, which outnumber EVs, overlap in size range with EVs, and could be falsely identified as EVs [10].

To standardize flow cytometry measurements, the measured fluorescence and light scattering signals require calibration to convert arbitrary units into standard units. For example, the arbitrary units of fluorescence intensity can be related to the standard unit molecules of equivalent soluble fluorochrome (MESF) [11,12]. The arbitrary units of light scattering intensity can be related to the diameter of EVs in nm. Calibrated fluorescence and light scattering signals allow to express EV concentrations within similar detection ranges.

Calibration requires stable, traceably characterized certified reference materials (RMs) with optical signal levels similar to EVs. Please note that RMs can be divided into (i) quality control materials and (ii) certified RMs [13,14]. With certified RM, we mean a sample containing reference particles, of which the physical property that is intended for calibration purposes is homogeneous, metrologically traceably characterized, and stable. With traceable, we mean that the measurement results of the property intended for calibration (eg, particle size) can be related to the International System of Units (SI) through an unbroken chain of comparisons with known uncertainties [[15], [16], [17]]. Please note that according to this definition, neither a stable sample with polydisperse EVs nor a monodisperse sample characterized by nontraceable detection techniques is a certified RM but is a quality control material. Currently available certified RMs are orders of magnitudes brighter in fluorescence and light scattering signals than EVs, and hence, no suitable certified RMs exist for calibrating flow rate, fluorescence, and light scatter of FCMs.

The mentioned challenges and the lack of suitable certified RMs for EV flow cytometry research have led to the 18HLT01 Metrological characterisation of microvesicles from body fluids as noninvasive diagnostic biomarkers (METVES) II (https://www.metves.eu). METVES II develops an infrastructure to standardize EV concentration measurements to enable multicenter studies on EVs. METVES II is developing certified RMs and procedures to calibrate all aspects of an FCM such that different FCMs can determine the concentration of EVs within the same fluorescence and light scattering range [6,11]. To validate whether the developed RMs and procedures indeed improve standardization, METVES II organized an interlaboratory comparison study wherein >20 FCMs are calibrated in order to determine the concentration of EVs in an EV-containing test sample within comparable detection ranges.

In a previous interlaboratory comparison study, aliquots of unstained frozen plasma samples were distributed as an EV-containing test sample. Participants had to fluorescently stain the plasma EVs themselves, which led to preanalytical and interuser variations in the measurement results [18].

Our aim was to develop a plasma EV test sample (PEVTES) that i) resembles subcellular particles in plasma, ii) is ready-to-use, iii) is flow cytometry–compatible, and iv) is stable. By stable, we mean that the concentration of EVs within a certain fluorescence or light scattering intensity range does not change over a time period corresponding to the intended purpose of the sample, such as an interlaboratory comparison study or quality control. In this article, we provide an overview of how to achieve each of the beforementioned aims (i-iv) and how to develop a PEVTES. The developed PEVTES is being used in an upcoming METVES II-organized interlaboratory comparison study to validate developed certified RMs and procedures.

## 2 Methods

### 2.1 Study design

**Figure 1:**
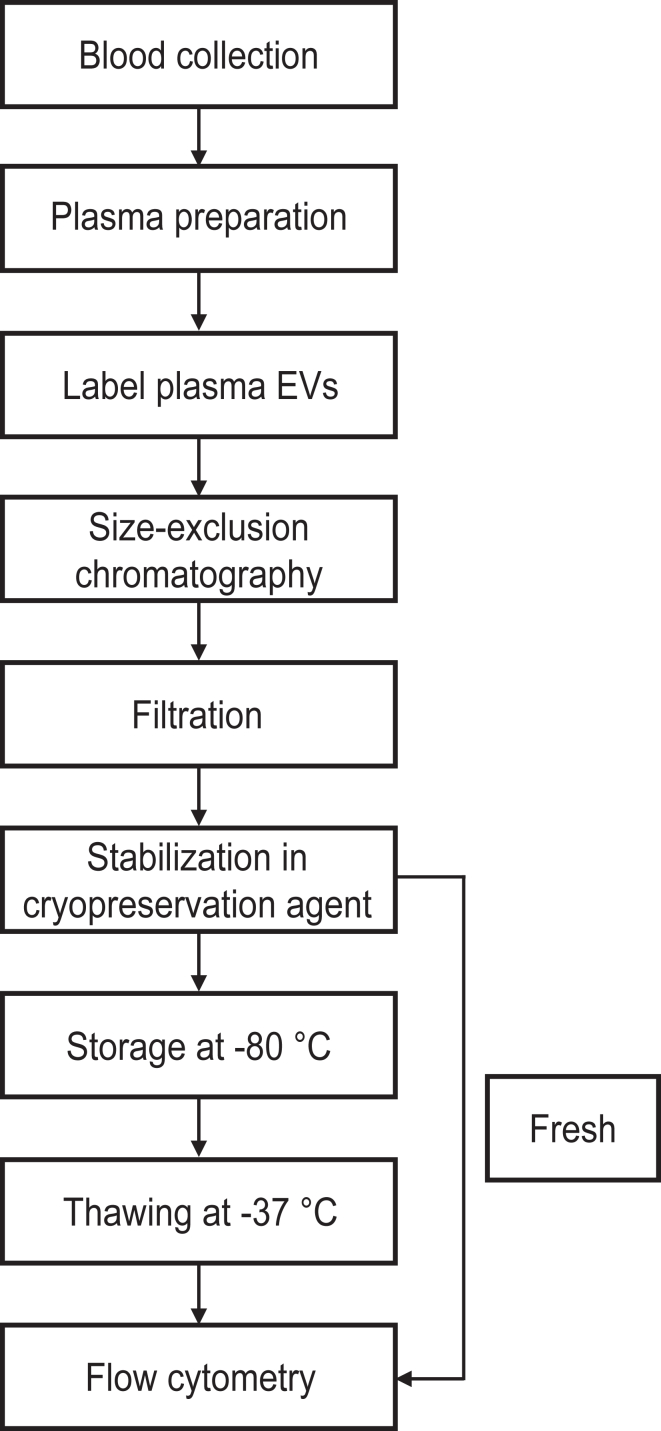
Experimental workflow to develop the plasma extracellular vesicle (EV) test sample (PEVTES). Schematic overview of the different steps involved in preparing the PEVTES. The steps include collection and preparation of human plasma, staining procedure, size-exclusion chromatography, removal of residual platelets with a 0.8-μm pore-sized polycarbonate filter, and stabilization of the PEVTES by adding different cryopreservation agents. Samples were measured either fresh or after 1, 3, 6, and 12 months of storage at −80 °C with a calibrated flow cytometer (Apogee A60-Micro, Apogee Flow Systems). To prepare the PEVTES + bovine serum albumin (BSA) sample for Figure 4, BSA (f.c. 0.5%; Sigma Aldrich) was added to the sample.

The aim of the study was to develop a PEVTES. The PEVTES should i) resemble subcellular particles in plasma because plasma is the most widely studied biofluid for EV biomarker research [19]. Therefore, plasma was chosen as a starting material. To be ii) ready-to-use, EVs present in plasma were immunofluorescently stained with antibodies against cell type–specific proteins. This makes the PEVTES a prestained sample. To ensure that the PEVTES iii) is flow cytometry–compatible, swarm detection of particles below the detection limit was minimized and unbound reagents were removed by size-exclusion chromatography (SEC). Furthermore, discoid-shaped residual platelets and tubular particles [8] were removed by filtration. Removing non-spherical particles is important because Mie theory, which relates scatter signals to particle diameter and refractive index, only applies to spherical particles [20]. To iv) stably store the samples, different cryopreservation agents were evaluated. To evaluate stability, the concentration of stained EVs was measured before freezing and after 1, 3, 6, and 12 months of storage at −80 °C with an FCM, of which the flow rate, fluorescence signals, and light scattering signals were calibrated. A schematic overview of the procedure can be found in Figure 1.

### 2.2 Preparation of the plasma EV test sample (PEVTES)

#### 2.2.1 Preparation of cell-depleted plasma

The collection of blood was performed according to the guidelines of the medical ethical committee of Amsterdam Medical Centre, University of Amsterdam (W18_391#18.450). Blood was collected from 3 healthy and overnight fasting individuals who provided informed consent and denied having a disease and/or using medication. Venous blood was collected using a 21-gauge needle (368607, Becton Dickinson [BD] Biosciences), and the first 3.5 mL of blood was discarded. Three tubes of EDTA blood [21] (6 mL, 9203871, BD Biosciences) were collected per donor, mixed gently with the anticoagulant, and processed within 15 minutes.

To prepare plasma, whole blood was centrifuged at 2500× *g* for 15 minutes at 20 °C, acceleration speed 9, and deceleration speed 1 using a Rotina 380 R equipped with a swing-out rotor and radius of 155 mm (Hettich Zentrifugen). Plasma was collected 10 mm (determined with a Lego brick) above the buffy coat using a plastic Pasteur pipette (86.1171.001, SARSTEDT) and transferred into a new 15-mL polypropylene centrifuge tube (62.9924272, SARSTEDT). Subsequently, the plasma was centrifuged at the same settings used for whole blood. Afterward, plasma was collected to 10 mm above the pellet to reduce platelet contamination and transferred into a new 15-mL polypropylene centrifuge tube (62.9924272, SARSTEDT). Next, plasma was pooled, mixed gently, and transferred to 1.5-mL low protein binding Eppendorf tubes (616201, Greiner Bio-One B.V.).

#### 2.2.2 Staining EVs for flow cytometry

To measure the concentration of platelet-derived (CD61-allophycocyanin [APC]), erythrocyte-derived (CD235a-phycoerythrin [PE]), and lactadherin-binding (lactadherin-fluorescein isothiocyanate [FITC]) EVs, plasma EVs were immunofluorescently stained. Before staining, aggregates present in the antibodies and lactadherin reagents were removed by centrifugation at 18,890× *g* for 5 minutes at 20 °C. The supernatant minus 10 μL of the starting volume was collected and used for staining. EVs were stained with antihuman CD61-APC antibody (17-0619-42; VI-PL2; final concentration [f.c.], 8.33 μg/mL; eBioscience), antihuman CD235a-PE antibody (R7078; JC159; f.c., 100 μg/mL; Dako), and lactadherin-FITC (BLAC-FITC; f.c., 41.5 μg/mL; Haematologic Technologies), mouse immunoglobulin (Ig)G_1_-APC (554681, MPOC-21, f.c. matched to CD61-APC; BD Biosciences), or IgG_1_-PE (345816, X40, f.c. matched to CD235a-PE; BD Biosciences). Furthermore, 5 mL of cell-depleted plasma was incubated with a combination of either i) 687.5 μL CD61-APC and 687.5 μL CD235a-PE, ii) 687.5 μL CD61-APC and 687.5 μL lactadherin-FITC, or iii) IgG_1_ isotype controls at matching concentrations and incubated for 2 hours at room temperature in the dark.

#### 2.2.3 Size-exclusion chromatography

Next, to separate EVs from unbound dye, soluble proteins, and reduce lipoprotein particles, SEC was performed (qEVsingle/70 nm1004125; Izon Science). Therefore, 1 mL of plasma containing the prestained EVs was loaded on each washed SEC column, followed by elution with Dulbecco’s phosphate-buffered saline (dPBS; 21-031-CVR, Corning). The first 3.5 mL eluate containing the void volume was discarded, after which the 1-mL fraction containing most EVs was collected and pooled.

#### 2.2.4 Platelet removal with polycarbonate filters

To remove remaining platelets from plasma, plasma was filtered using a 0.8-μm pore-size polycarbonate membrane filter (ATTP02500, Isopore, Merck Millipore) with a diameter of 25 mm. Typically, this step reduces the residual platelet concentration 1.5 × 10^2^-fold [22].

#### 2.2.5 Stabilization

**Figure 4:**
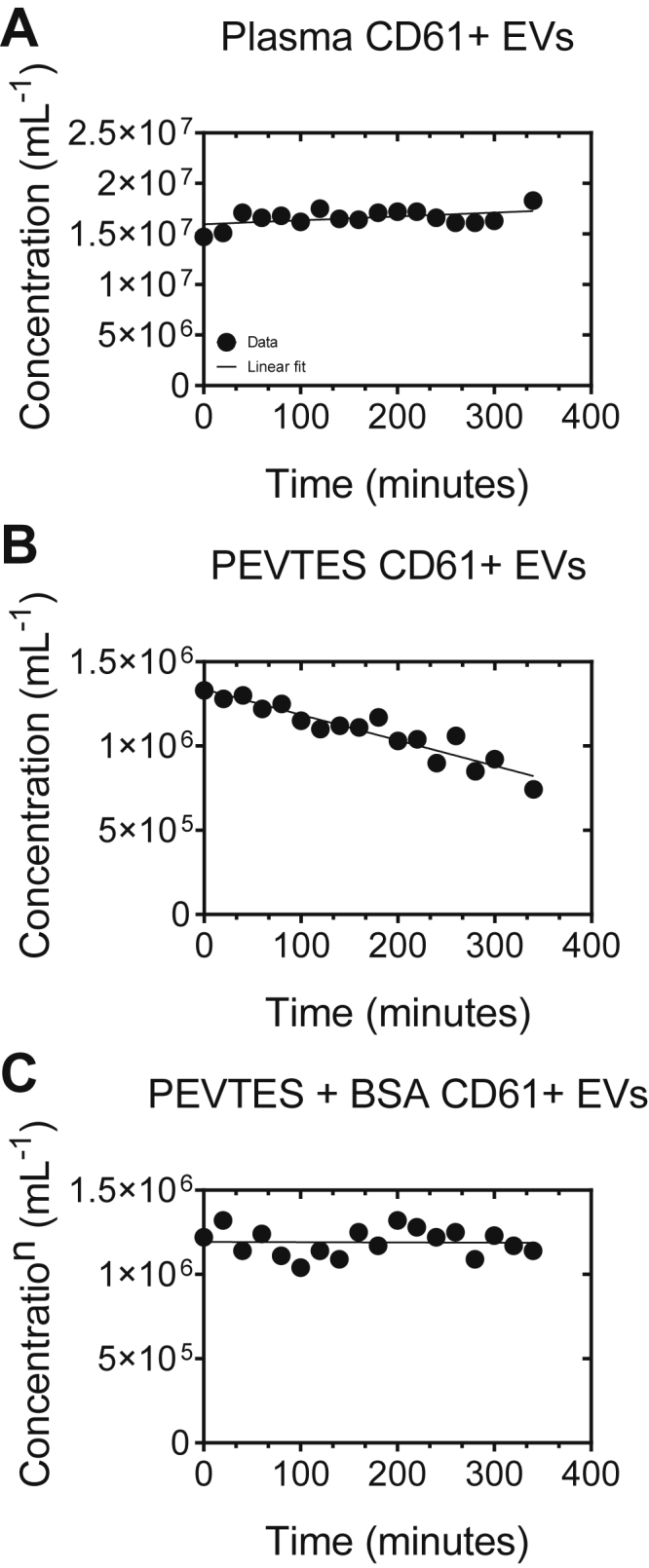
Bench stability of the plasma EV test sample (PEVTES) compared with human plasma. We found that the bench stability of the developed PEVTES decreased with measurement time. Therefore, we developed a procedure to stabilize the developed PEVTES by supplementing it with additional protein such as Bovine Serum Albumin (BSA), called stable PEVTES. Samples were measured with a calibrated flow cytometer (Apogee A60-Micro, Apogee Flow Systems). Data is presented as EV concentration (mL^−1^) versus measurement time (minutes). Data (symbols) have been fitted with a linear function (lines), resulting in a slope of 3839 and a *x*-intercept of −4157 for plasma, a slope of −1515 and *x*-intercept of 882 for the PEVTES, and a slope of −13 and a *x*-intercept of 88871 for the PEVTES + BSA. To prepare the PEVTES + BSA sample (stable PEVTES), BSA (f.c. 0.5%, Sigma Aldrich) has been added. For details, please see Supplementary Material S3.

To improve the stability of the prestained and SEC-isolated EVs, the cryopreservation agents, dimethyl sulfoxide (DMSO), glycerol, and trehalose, were selected based on the literature and tested [[23], [24], [25], [26], [27], [28]]. The optimal concentration of each cryopreservation agent for long-term stability was investigated in preliminary experiments (data not shown). The PEVTES was diluted 2× in either 20% DMSO (1.02931.500; f.c., 10%; Merck Millipore), 40% glycerol (1.37028.1000; f.c. 20%; Merck Millipore) or 1 M D (+)-trehalose dihydrate (T9531; f.c., 0.5 M; Sigma Aldrich). To prepare the PEVTES + bovine serum albumin (BSA) sample for Figure 4, BSA (A9647; f.c., 0.5%; Sigma Aldrich) was added to the sample.

### 2.3 Storage and thawing

Samples were frozen in liquid nitrogen and stored at −80 °C. Frozen samples were thawed in a water bath at 37 °C for 1 minute before use and kept at room temperature prior to measurements.

### 2.4 Procedural controls

Besides buffer-only and isotype controls, procedural controls were included to confirm the absence of particles introduced by the sample preparation procedure [29]. As a procedural control, the entire PEVTES procedure was applied to dPBS instead of cell-depleted plasma as a starting material.

### 2.5 Flow cytometry

The concentration of EVs was measured with a calibrated FCM (A60-Micro, Apogee Flow Systems), using settings optimized for detection of EVs. A full description of the FCM configuration, operating conditions, and data analysis can be found in the MIFlowCyt-EV (Supplementary Material S1).

All samples were measured in triplicate and procedural controls in duplicate in 96-well plates (655101, Greiner Bio-One B.V.) Prior to measurement, samples were prediluted in dPBS to event rates below 5000/s to further prevent swarm [30]. Samples were measured for 120 seconds at a flow rate of 3.0 μL/minute. The trigger detector was side scattering (SSC) operating at 405-nm illumination wavelength and the trigger threshold corresponds to a SSC cross-section of 10 nm^2^. The EV concentration in this experiment describes the number of particles that exceed the side scatter threshold with a diameter >200 nm as determined by Flow-SR [10] and that are positive at the relevant fluorescent detectors.

To exclude that variations in sensitivity of the FCM over time affect the measured EV concentrations, we (1) calibrated the fluorescence and scatter detectors at all the measurement days, (2) determined the lower detection limit of the scattering detector and the fluorescence detectors for all the measurement days, and (3) applied a lower gate to the scatter (10.15 nm^2^) and fluorescence detectors (185 APC MESF, 400 FITC MESF and 123 PE MESF) that is equal to the lower detection limit at the least sensitive measurement day over the time course of 12 months. Here, with the sensitivity of the scatter detector, we mean the SSC intensity corresponding to the trigger threshold, and with the sensitivity of the fluorescence detectors, we mean the fluorescence intensity that differentiates positively stained particles from the background fluorescence.

Data analysis was performed by custom-built software (MATLAB R2020b, MathWorks) to automate data calibration and data processing. Graphs were made with Prism 8.0 (GraphPad) and Adobe Illustrator (V 26.2.1, Adobe Inc).

## 3 Results

### 3.1 Plasma EV test sample resembles human plasma

**Figure 2:**
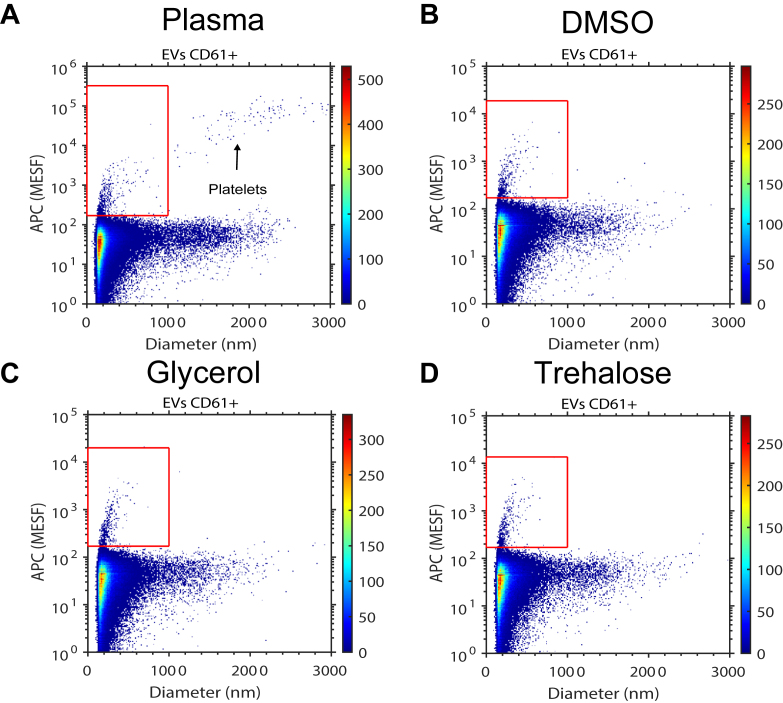
The plasma EV test sample (PEVTES) resembles human plasma. Scatter plots showing the diameter (nm) versus fluorescence intensity (allophycocyanin (APC) molecules of equivalent soluble fluorochrome (MESF) of (A) prestained human plasma, and (B–D) freeze-thawed plasma EV test sample (PEVTES) stored in (B) dimethyl sulfoxide (DMSO), (C) glycerol or (D) trehalose measured with a calibrated flow cytometer (Apogee A60-Micro, Apogee Flow Systems). The red gate shows the population of immuno-fluorescently stained (platelet-derived) EVs which can be distinguished from the background based on the scatter signal. Please note that Figure 2A includes platelets, indicated by an arrow within the red gate, while the PEVTES does not contain detectable levels of remaining platelets. For details, please see Supplementary Material S2.

To investigate if the developed PEVTES resembles immunofluorescently stained EVs in normal human plasma as measured on an FCM, we measured and compared immunofluorescently stained EVs from freeze-thawed pooled plasma and the PEVTES by flow cytometry. Figure 2A–D show scatter plots of the fluorescence intensity versus particle diameter of a CD61-APC-stained human plasma sample and a PEVTES after a single freeze-thaw cycle, respectively. The fluorescence signals distinguish CD61-APC–stained EVs (red gate) from the background. Both the CD61-APC–stained EVs and (unstained) non-EV particles in the PEVTES resemble those present in human plasma. Furthermore, the PEVTES does not contain detectable concentrations of platelets, which confirms that the procedure to remove platelets by filtration after SEC is effective. As the fluorescence signals vs the diameter of the PEVTES resembles EVs present in human plasma, we show that it is feasible to prepare and store stained plasma EVs.

### 3.2 Stability of the plasma EV test sample during storage

During storage at −80°C, the concentration and staining of EVs may be affected by the freezing and thawing process itself and/or by the storage time. Both processes are known to cause EV loss [31,32]. With the aim to investigate these processes independently, we measured EV concentrations freshly and at 4 time-points after storage.

**Figure 3:**
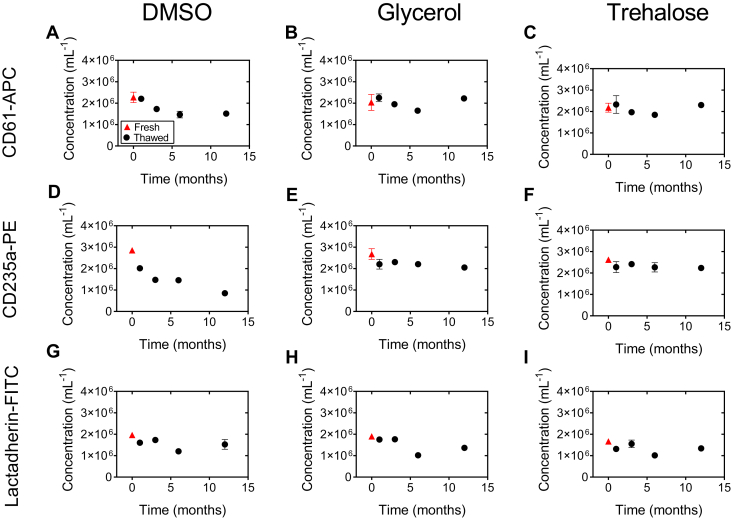
Plasma EV test sample (PEVTES) stability during storage. Concentration of platelet (CD61+, A–C), erythrocyte (CD235a+, D–F) or lactadherin+ (G–I), immuno-fluorescently stained EVs stored in dimethyl sulfoxide (DMSO; 10% f.c.), glycerol (20% f.c.) or trehalose (0.5 M f.c.) at −80 °C for 12 months. Samples were measured fresh (red triangle), after freeze thawing after 1, 3, 6 and 12 (black circle) months of storage with a calibrated flow cytometer (Apogee A60-Micro, Apogee Flow Systems). Data are presented as mean ± standard deviation EV concentration (mL^−1^) versus the storage time (months). For details, please see Supplementary Material S1.

Figure 3 shows the measured EV concentrations in the PEVTES during storage in DMSO, glycerol, and trehalose for up to 12 months at −80 °C. Compared with fresh starting material, which does not involve a freeze-thaw cycle, the measured concentration of CD61+ EVs decreased 33% when stored in DMSO for 12 months, increased 9% when stored in glycerol and increased 6% when stored in trehalose, Figures 3A–C, respectively. The concentration of CD235a+ EVs shown in Figures 3D–F decreased about 70% when stored in DMSO, 23% when stored in glycerol, and 15% when stored in trehalose compared with the fresh starting material, respectively. The concentration of lactadherin+ EVs shown in Figures 3G–I decreased 23% when stored in DMSO, 28% when stored in glycerol, and 19% when stored in trehalose, respectively.

We also compared the EV concentration in the PEVTES after 12 months of storage to 1 month of storage. Please note that both 12 months of storage and 1 month of storage include a single freeze-thaw cycle, and therefore, the most prominent variable is the storage time itself rather than the influence of freeze-thawing on the sample. The measured CD61^+^ EV concentration decreased 31% when stored in DMSO, 1% when stored in glycerol, and 1% when stored in trehalose, as shown in Figure 3A–C. The measured CD235a^+^ EV concentration stored for 12 months decreased 58% when samples were stored in DMSO, 7% when stored in glycerol, and 2% when stored in trehalose, compared with 1 month of storage at −80 °C, as shown in Figure 3D–F. Furthermore, the measured lactadherin^+^ EV concentration stored for 12 months decreased 5% when samples were stored in DMSO and 22% when stored in glycerol and increased 2% when stored in trehalose, compared with 1 month of storage at −80 °C, as shown in Figure 3G–I, respectively.

All in all, we show that the developed PEVTES can be stored stably for at least 12 months at −80 °C in the presence of a cryopreservation agent. Furthermore, we show that trehalose is the most suitable cryopreservation agent for our PEVTES.

### 3.3 Plasma EV test sample bench stability

Figure 4 shows the bench stability of the PEVTES. With bench stability, we mean the stability of the test sample after thawing when stored at room temperature. Figure 4A shows that the concentration of CD61^+^ EVs, over time in plasma, increased by 25% (coefficient of variation [CV], 5%), which we attribute to evaporation of the medium. Please note that the samples were not covered during measurement. This result emphasizes the relevance of cooling or covering the sample before measurement. Figure 4B shows that the concentration of CD61^+^ EVs in the PEVTES decreased 44% (CV, 15%) during the measurement time of 340 minutes at room temperature. We attribute the decrease in particle concentration over time to the adhesion of EVs to the plastic of the well plates in the absence of proteins [33]. Therefore, we developed a procedure to stabilize the developed PEVTES by adding BSA. Similarly, to plasma, Figure 4C shows a decrease in the CD61^+^ EV concentration in the PEVTES with BSA over 340 minutes (7% decrease; CV, 7%). This shows that the addition of BSA prevents adhesion of EVs to the surface of the well plates.

Taken together, the developed procedure is capable of producing prestained human plasma EVs that are stable upon storage at −80 °C for at least 12 months. Please note that prestained means that the PEVTES is a ready-to-use sample, which only requires dilution prior to measurement and no additional staining step.

## 4 Discussion and Conclusion

Although the concentration of EVs in body fluids may provide novel disease biomarkers, the measured EV concentrations of any given EV-containing sample are still incomparable between FCMs. To achieve comparability, new certified RMs with physical properties resembling EVs and reference procedures are being developed in the METVES II project. Validation of new certified RMs requires testing on different FCMs using an EV-containing test sample because the variation in the measured concentrations of EVs should not be caused by the EV-containing test sample itself. The last conducted interlaboratory comparison study revealed the need for such a ready-to-use EV-containing test sample [18]. Therefore, we developed the PEVTES that i) resembles subcellular particles in plasma, ii) is ready-to-use, iii) is flow cytometry–compatible, and iv) is stable during storage.

The Table shows an overview of currently available test samples for EV flow cytometry, which include plasma, recombinant EVs (rEVs) [34], and engineered retroviruses [35]. Our results show that within the detection range of an FCM, EVs and non-EV particles in the PEVTES resemble particles found in human plasma (Figure 2A–D). Furthermore, whereas both EVs present in the PEVTES and in plasma are polydisperse, rEVs are only partly polydisperse and engineered retrovirus samples are monodisperse. The PEVTES also contain non-EV particles in the relevant size ranges, which both rEVs and engineered retrovirus do not. compared with human plasma, rEVs, and engineered retroviruses, only the PEVTES is ready-to-use because EVs present in the sample are stained. For the labels used, we show that it is feasible to freeze stained samples because the fluorescence signal of the PEVTES sample is similar to the fluorescence signal of EVs present in immunofluorescently stained plasma (Figure 2). Please note that only a fraction of EVs present in the PEVTES is stained, and therefore, we are only detecting cell-type–specific EVs and not all EVs present in the developed sample.

**Table:** Overview of a selection of available quality control samples for EV flow cytometry.

Property	PEVTES	Frozen plasma	Recombinant EVs [34]	Engineered retrovirus [35]
Stable during storage	Yes	Probably	Yes	Yes
Stable after storage	Yes	Probably	Yes	Yes
Ready-to-use	Yes	No	No	No
prestained	Yes	No	No	No
Resembles subcellular particles in plasma	Yes	Yes	No	No
Polydisperse	Yes	Yes	Partly	No
Non-EV particles	Yes	Yes	No	No
Unbound reagents	No	n/a	n/a	n/a
Cells absent	Yes	No	n/a	n/a
Risk of swarm detection	Mitigated	Yes	n/a	n/a

EV, extracellular vesicle; PEVTES, plasma extracellular vesicle test sample.

To develop a flow cytometry–compatible sample, we decreased the concentration of particles <70 nm to reduce swarm detection by SEC, which will also deplete the bulk of soluble proteins, including unbound antibodies. After SEC, we applied a 0.8-μm pore-size polycarbonate membrane filter to remove particles with a diameter >800 nm, which include discoid-shaped platelets and tubular particles [8]. By removing the discoid and nonspherical particles, we ensure the validity of Mie theory to calibrate light scattering signals. While frozen human plasma still contains cells [22], PEVTES presents a cell-depleted sample. By removal of platelets before freezing, cell fragmentation can be excluded.

Freezing may cause membrane damage and loss of EV function [36]. Generally, rEVs, engineered retrovirus samples, and PEVTESs are all stable during and after storage, whereas this might not be the case for frozen human plasma [37]. To prepare a stable sample, different cryopreservation agents (DMSO [23,27], glycerol [24,25], and trehalose [25,26,28]) were added to the PEVTES sample before freezing. The use of cryopreservation agents is a commonly accepted procedure to prevent osmotic damage and preserve protein stability [38].

Generally, we showed that the 3 different cryopreservation agents had different degrees of stabilizing effectiveness, expressed as the percentage decrease in the plasma-EV concentration over time (Figure 4). DMSO and glycerol, both penetrating cryopreservation agents, had lower stabilizing effectiveness than trehalose [24]. Nonpenetrating cryopreservation agents, such as trehalose and other sugars, may represent more biocompatible cryopreservation agents [39]. In our experiments, trehalose best preserved prestained EVs present in the PEVTES as measured by a minimal decrease in EV concentration over 12 months of storage at −80°C.

Besides storage stability, we found that the bench stability of the PEVTES decreased with measurement time (Figure 4). First, we observed that the total particle concentration and CD61^+^ EV in human plasma increased with measurement time. The increase in particles can be attributed to evaporation of the surrounding medium (Supplementary Material S3, Supplementary Figures S3.1 and S4.1). [40,41] Furthermore, we observed a decrease in CD61^+^ EV and total particle concentration (Supplementary Material S3, Supplementary Figure S3.1). We attributed the loss of EVs to adherence of EVs to the surface of the 96-well plates. In line with our observation, other studies also warned about possible adherence of EVs to surfaces [33,36,42]. Multiple studies suggested that the addition of a soluble protein, such as BSA, might reduce the loss of EVs to “normal” tubes (eg, Eppendorf) or the use of low-binding plastics. Evtushenko et al. [33] showed that particle loss could be reduced by 18% when the surface of plastic tubes was blocked with BSA. In line with our first observation that the total particle concentration in human plasma increased with measurement time, we observed a similar trend for the PEVTES sample containing BSA. Evaporation and adhesion of EVs to plastic are processes that may occur concurrently.

In sum, we can conclude that the prestained EVs present in the PEVTES are stable during storage for at least 12 months but require the addition of BSA to improve bench stability. All in all, the new and improved PEVTES is stable after storage for at least 12 months at −80 °C and for 6 hours on bench.

The developed PEVTES has multiple potential applications, including a quality control sample for monitoring day-to-day variability of instruments measuring EV properties such as light scattering intensity, fluorescence intensity, size, number concentration, and refractive index. Furthermore, the PEVTES may be useful to optimize settings of instruments measuring EV properties or to develop new techniques measuring EV properties.

Taken together, the developed PEVTES i) resembles subcellular particles in plasma, ii) is ready-to-use, iii) is flow cytometry–compatible, and iv) is stable. The developed PEVTES will be used in an interlaboratory comparison study to validate newly developed certified RMs and procedures. In the future, traceable characterization of the size distribution, number concentration, fluorescence intensity, and refractive index of EVs in the PEVTES is planned. This will be the first time in the field of EV research that all these characteristics are traceably measured for a biological test sample. Together with EV-dedicated certified RMs, the developed PEVTES sample is an essential part of the infrastructure needed to facilitate multicenter studies in the field of EV research in the future.
