# Supplementary material for: Plasma extracellular vesicle test sample to standardize flow cytometry measurements
Source: Res Pract Thromb Haemost. 2023 May 18;7(4):100181. doi: 10.1016/j.rpth.2023.100181 (PMC10394550; doi:10.1016/j.rpth.2023.100181)
Supplement: Supporting Information 1 [file mmc1.docx]

Supporting Information 1: MIFlowCyt-EV checklist of “Stable, ready-to-use test sample containing stained extracellular vesicles from human plasma to standardize flow cytometry measurements”

1. **Flow cytometry**
   1. **Experimental design**

Our aim was to develop a plasma extracellular vesicle (EV) test sample (PEVTES) that i) resembles subcellular particles in plasma, ii) is ready-to-use, iii) is flow cytometry compatible, and iv) is stable. To evaluate PEVTES stability, the concentration of stained EVs (Cluster of differentiate (CD), CD61-allophycocyanin (APC)+, CD235a- phycoerythrin (PE)+, Fluorescein isothiocyanate (FITC)+) was measured before freezing and after 1, 3, 6, and 12 months of storage at -80°C with a flow cytometer (A60-Micro, Apogee Flow Systems) of which the flow rate, fluorescence signals and light scattering signals were calibrated.

Pre-analytical variables, such as blood collection, plasma and PEVTES preparation, are reported in the manuscript.

All samples were measured during a period of 12 months at different time points of storage. All samples were measured using an autosampler, which enables the measurement of samples in a 96-well plate. The 96-well plate contained buffer-only controls, and isotype controls corresponding to the labels chosen for those experiments. Additionally, procedural controls were included to confirm the absence of particles introduced by the sample preparation procedure. As a procedural control, the entire PEVTES procedure was applied to Dulbecco’s Phosphate-buffered saline (dPBS) instead of cell-depleted plasma as a starting material.

Flow rate, fluorescence, and light scatter calibrations were performed on the day of the experiments. To automatically process data, apply calibrations, determine and apply gates, generate reports with scatter plots and generate data summaries, custom-build software (MATLAB R2018b, Mathworks) was used.

The FCM underwent maintenance after the study was running for 6 months.To exclude that variations in sensitivity of the FCM over time affect the measured EV concentrations, we (1) calibrated the fluorescence and scatter detectors at all measurement days, (2) determined the lower detection limit of the scattering detector and the fluorescence detectors for all the measurement days, and (3) applied a lower gate to the scatter (10.15 nm^2^) and fluorescence detectors (185 APC molecules of equivalent soluble fluorophore (MESF), 400 FITC MESF and 123 PE MESF) that is equal to the lower detection limit at the least sensitive measurement day over the time course of 12 months

- 1. **Sample dilutions**

The particle concentration in plasma differs between individuals, and therefore samples require different dilutions to avoid swarm detection [1] and to achieve statistically significant counts. Previously, a procedure to estimate to optimal sample dilution has been developed at our lab [2]. In summary Buntsma et al. showed that for our flow cytometer and settings used, a count rate ≤ 5.0∙10^3^ events per second unlikely results in swarm detection. To find the dilution resulting in a count rate ≤5.0∙10^3^ events per second, we diluted PEVTES 25-fold in dPBS (21-031-CV, Corning) and measured the total concentration of particles for 120 seconds. By diluting PEVTES 25-fold, all samples had a count rate ≤5.0∙10^3^ events per second.

- 1. **EV staining**

To measure the concentration of platelet-derived (CD61- APC), erythrocyte-derived (CD235a- PE), and lactadherin-binding (lactadherin-FITC) plasma EVs in PEVTES were immuno-fluorescently stained in the fresh sample. Before staining, aggregates present in the antibodies and lactadherin reagents were removed by centrifugation at 18,890 g for 5 minutes at 20 °C. The supernatant minus 10 μL of the starting volume was collected and used for staining. EVs were stained with anti-human CD61-APC antibody (17-0619-42, VI-PL2, final concentration (f.c.) 8.33 μg/mL; eBioscience) anti-human CD235a-PE antibody (R7078, JC159, f.c. 100 μg/mL; Dako) and lactadherin-FITC (BLAC-FITC, f.c. 41.5 μg/mL; Haematologic technologies), mouse IgG1- APC (554681, MPOC-21, f.c. matched to CD61-APC; BD Biosciences), or IgG1-PE (345816, X40, f.c. matched to CD235a-PE; BD Biosciences). Furthermore, 5 mL of cell-depleted plasma was incubated with either a combination of i) 687.5 μL CD61-APC and 687.5 μL CD235a-PE, or ii) 687.5 μL CD61-APC and 687.5 μL lactadherin-FITC, or iii) IgG1 isotype controls. An overview of the staining reagents can be found in Table S1.1.

- 1. **Buffer-only control**

Each 96-well plate contained at least 1 well with dPBS, which was measured with the same flow cytometer and acquisition settings as all other samples. The mean count rate for all time points was 22 events per second, which is lower than the target count rate (2.5-5.0∙10^3^ events per second) for plasma samples. Next to dPBS each 96-well plate contained 1 well of cryopreservation agent (DMSO, glycerol and trehalose), which was used to stabilize samples. The mean count rate for all time points in DMSO buffer was 31 events per second, 25 events per second in glycerol, and 31 events per second in trehalose, which is lower than the target count rate (2.5-5.0∙10^3^ events per second) for plasma samples.

- 1. **Procedural control and buffer with reagents control**

Besides buffer-only and isotype controls, procedural controls were included to confirm the absence of particles introduced by the sample preparation procedure [3]. As a procedural control, the entire PEVTES procedure was applied to dPBS instead of cell-depleted plasma as a starting material. Additionally, the procedural control served as buffer with reagent controls as buffer (DMSO, glycerol, trehalose) and in combination with each reagent (Table S1.1) was measured. Procedural controls were measured fresh, after 1, 3 and 6 months of storage at -80°C with the same flow cytometer and acquisition settings as all samples.

In DMSO combined with CD61-APC and CD235a-PE samples the average events per second for all 4 time points was 43. In glycerol combined with CD61-APC and CD235a-PE samples the average events per second for all 4 time points was 58. To investigate whether the counts caused by CD61-APC and CD235a-PE affected the reported results, we applied the same calibrations and gates to CD61-APC and CD235a-PE in all buffers as to the plasma samples stained with the corresponding antibody. On average based on all 4 time points, we obtained 7-8 CD61-APC+ events in DMSO, glycerol, and trehalose which is acceptable compared to 416 CD61-APC+ EVs in DMSO stored PEVTES (average 6 time points), 486 CD61-APC+ EVs in glycerol stored PEVTES, and 512 CD61-APC+ EVs in trehalose stored PEVTES. On average based on all 4 time points, we obtained 7-8 CD235a-PE+ events in DMSO, glycerol, and trehalose which is acceptable compared to 359 CD235a-PE+ EVs in DMSO stored PEVTES (average 6 time points), 551 CD235a-PE+ EVs in glycerol stored PEVTES, and 569 CD235a-PE+ EVs in trehalose stored PEVTES. On average based on all 4 time points, we obtained 13-19 lactadherin+ events in DMSO, glycerol, and trehalose which is acceptable compared to 397 lactadherin-FITC+ EVs in DMSO stored PEVTES (average 6 time points), 375 CD61-APC+ EVs in glycerol stored PEVTES, and 333 CD61-APC+ EVs in trehalose stored PEVTES.

- 1. **Isotype controls**

Table S1.1 shows an overview of the used isotype controls. In all 3 cryopreservation agents (DMSO, glycerol,trehalose) measured at all time points (T0-T12), we obtained on average 15 IgG1-APC+ events and 98 IgG1-PE+ events with a diameter ≤1,000 nm per measurement during 120 seconds. For comparison, on average 521 CD61-APC+, and 655 CD235a-PE+ events with a diameter ≤1,000 nm, were obtained in the experiments for fresh PEVTES. The numbers are based on the average of EV counts per EV marker in freshly measured PEVTES for all 3 cryopreservation agents.

- 1. **Trigger channel and threshold**

Based on the buffer-only control (dPBS, 22 events per second), the acquisition software was set up to trigger at 14 arbitrary units SSC, which is equivalent to a side scattering cross section of 10 nm^2^ (Rosetta Calibration, Rosetta Calibration, v1.13 Exometry B.V.).

- 1. **Flow rate quantification**

On the measurement days, we used 110 nm FITC beads with a specified concentration (Apogee calibration beads, Apogee Flow Systems) to calibrate the flow rate of the A60-Micro. Figure S1.2 shows the measured flow rate at the A60-Micro versus the date that experiments were performed. The adjusted flow rate is 3.01 µL/min and the measured median flow rate is 3.07 µL/min. For all days, the measured flow rate was within 10% of the adjusted flow rate.

- 1. **Fluorescence calibration**

Daily calibration of the fluorescence detectors from arbitrary units (a.u.) to molecules of equivalent soluble fluorochrome (MESF) was accomplished by performing daily cross-calibrations using Rainbow beads (RCP-30-5A, Spherotech Inc.)

Rainbow beads were cross calibrated against MESF beads to assign calibrated fluorescence intensity values to each population. Cross calibration was accomplished by using MESF beads (2 µm Q-APC beads (2321-175, BD), Quantum^TM^ FITC-5 MESF Kit (555A, 13734, Bangs Laboratories), and SPHERO PE Calibration Particle Kit, 3.0 -3.4 µm (ECFP-F2-5K, AK01, Spherotech Inc.). Cross-calibration was performed twice due to maintenance of the instrument after 6 months, on 20.05.2020 and 15.07.2021 (Table S1.2).

Daily calibrations of the APC, FITC, and PE detectors were performed on 08.07.2020 (T0), 10.08.2020 (T1), 07.10.2020 (T3), 08.01.2021 (T6), and on 21.07.2021 (T12) (Table S1.3). For each measurement, we added fluorescent intensities in MESF to the flow cytometry data files by custom-build software (MATLAB R2018a) using following equation:

| $\text{I(MESF)}={10}^{a\cdot\log_{10} \text{I(a.u.)}+b}$ | Equation S1 |
| --- | --- |

where I, is the fluorescence intensity, and *a* and *b* are the slope and the intercept of the linear fits respectively, see Table S1.3.

- 1. **Light scatter calibration**

We used Rosetta Calibration (Exometry B.V.) to relate scatter measured by forward scattering (FSC) and side scattering (SSC) to the effective scattering cross section and diameter of EVs. Figure S4.1 shows print screens of the scatter calibrations. We modelled EVs as core-shell particles with a core refractive index of 1.38, a shell refractive index of 1.48, and a shell thickness of 6 nm. For each measurement, we added the FSC and SSC cross sections and EV diameters to the flow cytometry datafiles by custom-build software (MATLAB R2020b, MathWorks) The SSC trigger threshold corresponds to a side scattering cross section of 10 nm^2^.

- 1. **MIFlowCyt checklist**

The MIFlowCyt checklist is added to Table S1.4.

- 1. **EV number concentration**

The concentrations reported in the manuscript describe the number of particles (1) that exceeded the SSC threshold, corresponding to a side scattering cross section of 10 nm^2^, (2) that were collected during time intervals, for which the count rate was within 50% of the median count rate, (3) with a diameter <1,000 nm as measured by SSC after light scatter calibration (section 1.10) and (4) are positive for APC, FITC, or PE, per mL of plasma.

- 1. **Data sharing**

Data is available via: <https://figshare.com/projects/Plasma_EV_Test_sample_PEVTES_Supporting_Material_1/161248>

1. **References**

1 van der POL E, van GEMERT MJC, STURK A, NIEUWLAND R, van LEEUWEN TG. Single vs. swarm detection of microparticles and exosomes by flow cytometry. *Journal of Thrombosis and Haemostasis TA  - TT  -* 2012; **10**: 919–30.

2 MIFlowCyt-EV of “EDTA stabilizes the concentration of platelet-derived extracellular vesicles during blood collection and handling” 1 Flow cytometry.

3 Welsh JA, Van Der Pol E, Arkesteijn GJA, Bremer M, Brisson A, Coumans F, Dignat-George F, Duggan E, Ghiran I, Giebel B, Görgens A, Hendrix A, Lacroix R, Lannigan J, Libregts SFWM, Lozano-Andrés E, Morales-Kastresana A, Robert S, De Rond L, Tertel T, et al. MIFlowCyt-EV: a framework for standardized reporting of extracellular vesicle flow cytometry experiments. *J Extracell Vesicles* Taylor & Francis; 2020; **9**: 1713526.

**Figures and tables**

**Figure S1.1** Forward scatter and side scatter calibration of the A60-Micro by Rosetta Calibration (Exometry B.V.). To relate scatter to the diameter of EVs, we modelled EVs as core-shell particles with a core refractive index of 1.38, a shell refractive index of 1.48, and a shell thickness of 6 nm.

**Figure S1.2** shows the measured flow rate at the A60-Micro versus the date that experiments were performed. The adjusted flow rate is 3.01 µL/min and the measured median flow rate is 3.07 µL/min. For all days, the measured flow rate was within 10% of the adjusted flow rate.

**Table S1.1: Overview of staining reagents.**  Characteristics being measured, analyte, analyte detector, reporter, isotype, clone, concentration, manufacturer, catalog number and lot number of used staining reagents.

| **Characteristic**  **measured** | **Analyte** | **Analyte detector** | **Reporter** | **Isotype** | **Clone** | **Concentration during staining (µg mL^-1^)** | **Manufacturer** | **Catalog number** | **Lot number** |
| --- | --- | --- | --- | --- | --- | --- | --- | --- | --- |
| Integrin | Human  CD61 | Anti-human CD61 antibody | APC | IgG1 | VI-PL2 | 25 | eBioscience | 17-0619-42 | 2062626 |
| Glycoprotein | CD235a | Anti-human CD235a  antibody | PE | IgG1 | JC159 | 100 | Dako | R7078 | 20067598 |
| Glycoprotein | Lactadherin | Lactadherin | FITC | n.a. | n.a. | 41.5 | Haematologic Technologies | Blac-FITC | KK0122 |
| Affinity for Fc receptor | Fc receptor | IgG1 | APC | n.a. | MPOC-21 | 25 | BC Bioscience | 554681 | 7075605 |

APC: allophycocyanin; FITC: fluorescein isothiocyanate; IgG: Immunoglobulin G; PE: phycoerythrin.

**Table S1.2: Overview of fluorescence calibrations used for cross calibration of the Rainbow beads.** Rainbow beads were cross calibrated against molecules of equivalent soluble fluorochrome (MESF) beads to assign calibrated fluorescence intensity values to each population. MESF/ cross calibration was performed twice due to maintenance of the instrument after 6 months.

| **Fluorophore** | **Calibration date** | **Slope** | **Intercept** | **R^2^** |
| --- | --- | --- | --- | --- |
| APC | 20.05.2020 | 1.208527815 | -2.00233 | 0.9945 |
| FITC | 20.05.2020 | 1.229137277 | -1.48284 | 0.9987 |
| PE | 20.05.2020 | 1.033830724 | -1.61284 | 0.9994 |
| APC | 15.07.2021 | 1.130251614 | -1.82115 | 0.9977 |
| FITC | 15.07.2021 | 1.141311319 | -1.13165 | 0.9991 |
| PE | 15.07.2021 | 1.020647906 | -1.50569 | 0.9998 |

APC: allophycocyanin; FITC: fluorescein isothiocyanate; PE: phycoerythrin

**Table S1.3: Overview of daily fluorescence calibration based on Rainbow beads.** Shown are the daily fluorescence calibration based on Rainbow beads that were applied to the arbitrary unit flow cytometry data for each measurement time point of plasma EV test sample (PEVTES) including the slope, intercept and R^2^.

| **Fluorophore** | **Time point** | **Date** | **Slope** | **Intercept** | **R^2^** |
| --- | --- | --- | --- | --- | --- |
| APC | Fresh | 08.07.2020 | 1.1992 | -1.9894 | 1.0000 |
|  | 1 months of storage | 10.08.2020 | 1.2005 | -1.9266 | 1.0000 |
|  | 3 months of storage | 07.10.2020 | 1.2128 | -2.0178 | 1.0000 |
|  | 6 months of storage | 08.01.2021 | 1.1841 | -1.8936 | 1.0000 |
|  | 12 months of storage | 21.07.2021 | 1.0946 | -1.4537 | 0.9974 |
| FITC | Fresh | 08.07.2020 | 1.2266 | -1.5078 | 0.9947 |
|  | 1 months of storage | 10.08.2020 | 1.2339 | -1.4794 | 0.9949 |
|  | 3 months of storage | 07.10.2020 | 1.2247 | -1.4388 | 0.9950 |
|  | 6 months of storage | 08.01.2021 | 1.2284 | -1.5832 | 0.9937 |
|  | 12 months of storage | 21.07.2021 | 1.1280 | -1.0765 | 0.9999 |
| PE | Fresh | 08.07.2020 | 1.0560 | -1.6482 | 0.9999 |
|  | 1 months of storage | 10.08.2020 | 1.0400 | -1.6346 | 1.0000 |
|  | 3 months of storage | 07.10.2020 | 1.0356 | -1.6207 | 1.0000 |
|  | 6 months of storage | 08.01.2021 | 1.0329 | -1.5995 | 1.0000 |
|  | 12 months of storage | 21.07.2021 | 1.0190 | -1.5004 | 1.0000 |

APC: allophycocyanin; FITC: fluorescein isothiocyanate; PE: phycoerythrin

**Table S1.4. MIFlowCyt checklist.**

| **Requirement** | **Please Include Requested Information** |
| --- | --- |
| 1.1. Purpose | To evaluate PEVTES stability, the concentration of stained EVs (Cluster of differentiate (CD), CD61-APC+, CD235a-PE+, lactahderin-FITC+) was measured before freezing and after 1, 3, 6, and 12 months of storage at -80°C with a flow cytometer (A60-Micro, Apogee Flow Systems, Hemel Hempstead, UK) of which the flow rate, fluorescence signals and light scattering signals were calibrated. |
| 1.2. Keywords | Blood plasma, Calibration, Extracellular vesicles, Flow cytometry, Quality control, Reproducibility, Standardization, |
| 1.3. Experiment variables | Time of storage at -80°C. |
| 1.4. Organization name and address | Amsterdam UMC location University of Amsterdam  Meibergdreef 9  1105 AZ Amsterdam  The Netherlands |
| 1.5. Primary contact name and email address | Britta A. Bettin, [b.a.bettin@amsterdamumc.nl](mailto:b.a.bettin@amsterdamumc.nl) |
| 1.6. Date or time period of experiment | Experiments were conducted within 12 months, from July 2020 to July 2021. Experiments were conducted on the following dates: 08.07.2020, 10.08.2020, 07.10.2020, 08.01.2021, 21.07.2021. |
| 1.7. Conclusions | Different subtypes of EVs within the PEVTES can be stored stably for at least 12 months at -80 °C in the presence of a cryopreservation agent. Trehalose is the most suitable cryopreservation agent for the developed PEVTES. |
| 1.8. Quality control measures | All samples were measured using an autosampler, which facilitates subsequent measurements of samples in a 96-well plate (655101, Greiner Bio-One B.V.)The well plate contained buffer-only controls (section S1.4) and isotype controls (section S1.6).  Besides buffer-only and isotype controls, procedural controls were included to confirm the absence of particles introduced by the sample preparation procedure (section S1.5). As a procedural control, the entire PEVTES procedure was applied to dPBS instead of cell-depleted plasma as a starting material.  The flow rate was calibrated with Apogee Calibration beads (1493, Apogee Flow Systems, section S1.8). Fluorescence detectors were calibrated daily (section S1.9) via cross-calibration using Rainbow beads (RCP-30-5A, Spherotech Inc.). Cross calibration was accomplished by using MESF beads (2 µm Q-APC beads (2321-175, BD), Quantum^TM^ FITC-5 MESF Kit (555A, 13734, Bangs Laboratories), and SPHERO PE Calibration Particle Kit, 3.0 -3.4 µm (ECFP-F2-5K, AK01, Spherotech Inc.).  FSC and SSC were calibrated with Rosetta Calibration (v1.13, section S1.10). |
| 1.9 Other relevant experiment information | The entire experiment was measured over 12 months. |
| 2.1.1.1. Sample description | Freshly prepared plasma (section 2.1.1.2) from 3 healthy volunteer (section 2.1.1.3). |
| 2.1.1.2. Biological sample source description | Venous blood was collected using a 21-Gauge needle (368607, Becton Dickinson (BD) Biosciences), and the first 3.5 mL of blood was discarded. Three tubes of EDTA blood (6 mL, 9203871, BD Biosciences) were collected per donor, mixed gently with the anti-coagulant, and processed within 15 minutes. To prepare plasma, whole blood was centrifuged at 2,500 g, 15 minutes, 20 °C, acceleration speed 9, deceleration speed 1 using a Rotina 380 R equipped with a swing-out rotor and radius of 155 mm (Hettich Zentrifugen). Plasma was collected 10 mm (determined with a Lego brick) above the buffy coat using a plastic Pasteur pipette (86.1171.001, SARSTEDT), and transferred into a new 15-mL polypropylene centrifuge tube (62.9924272, SARSTEDT) Subsequently, the plasma was centrifuged at the same settings used for whole blood. Afterwards, plasma was collected to 10 mm above the pellet to reduce platelet contamination, transferred into a new 15-mL polypropylene centrifuge tube (62.9924272, SARSTEDT). Next, plasma was pooled, mixed gently and transferred to 1.5-mL low protein binding Eppendorf tubes (616201, Greiner Bio-One B.V.). |
| 2.1.1.3. Biological sample source organism description | Healthy human volunteer. |
| 2.2 Sample characteristics | Plasma is expected to contain detectable EVs, lipoproteins proteins, and platelets. |
| 2.3. Sample treatment description | Plasma EV test sample (PEVTES) were developed based on human plasma (section 2.1.1.2). After plasma collection plasma EVs were immuno-fluorescently stained with CD61- APC, CD235a-PE, and lactadherin-FITC (section S1.3, Table S.1.1). Next, to separate EVs from unbound dye, soluble proteins, and reduce lipoprotein particles, SEC was performed (qEVsingle/70 nm1004125; Izon Science). To remove remaining platelets from plasma, plasma was filtered using 0.8-µm pore-size polycarbonate membrane filter (ATTP02500, IsoporeTM, Merck Millipore) with a diameter of 25 mm. To improve the stability of the pre-labelled and SEC-isolated EVs, the cryopreservation agents, dimethyl sulfoxide (DMSO), glycerol and trehalose were selected based on literature and tested (20–25). The optimal concentration of each cryopreservation agent for long-term stability was investigated in preliminary experiments (data not shown). The PEVTES was diluted 2x in either 20% DMSO (1.02931.500, f.c. 10%; Merck Millipore), 40% glycerol (1.37028.1000, f.c. 20%; Merck Millipore) or 1 molar D (+)-Trehalose dihydrate (T9531, f.c. 0.5 M; Sigma Aldrich). |
| 2.4. Fluorescence reagent(s) description | Please see Table S1.1. |
| 3.1. Instrument manufacturer | Apogee Flow Systems |
| 3.2. Instrument model | A60-Micro |
| 3.3. Instrument configuration and settings | Samples were analysed for 120 seconds at a flow rate of 3.01 μL/min on an A60-Micro, equipped with a 405 nm laser (100 mW), 488 nm laser (100 mW) and 638 nm laser (100 mW).  The instrument underwent maintenance after 6 months and was upgraded from from 300uA/lm to 650uA/lm photocathode, and the 638 nm laser was upgraded to 180 mW (adjustable). The trigger threshold was set at SSC 14 arbitrary units, corresponding to a side scattering cross section of 10 nm^2^ (Rosetta Calibration, Exometry B.V.). For the first 6 months of the study the voltage for FSC and SSC were 380 V and 350 V, respectively. For all detectors, the peak height was analysed. APC signals were collected with the 638-D Red (Peak) detector (long pass 652 nm filter, PMT voltage 510 V). FITC signals were collected with the 488-Green (Peak) detector (525/50 nm band pass filter, PMT voltage 520 V). PE signals were collected with the 488-Orange (Peak) detector (575/30 nm band pass filter, PMT voltage 520 V). After maintenance, for the 12-month time point the voltages for FSC and SSC were 348 V and 350 V, respectively. The voltage for APC was 423 V, for FITC 476 V and for PE 459 V. |
| 4.1. List-mode data files | A summary of all flow cytometry scatter plots and gates applied are available via:  <https://doi.org/10.6084/m9.figshare.22210876.v1>; <https://doi.org/10.6084/m9.figshare.22210891.v1>; <https://doi.org/10.6084/m9.figshare.22210897.v1>; <https://doi.org/10.6084/m9.figshare.22210903.v1>;  <https://doi.org/10.6084/m9.figshare.22210927.v1>;  <https://doi.org/10.6084/m9.figshare.22210942.v1> . |
| 4.2. Compensation description | No compensation was required because no fluorophore combinations were used that have overlapping emission spectra. |
| 4.3. Data transformation details | Fluorescence detectors were calibrated daily (section S1.9) via cross-calibration using Rainbow beads (RCP-30-5A, Spherotech). Cross calibration was accomplished by using MESF beads (2 µm Q-APC beads (2321-175, BD), Quantum^TM^ FITC-5 MESF Kit (555A, 13734, Bangs Laboratories), and SPHERO PE Calibration Particle Kit, 3.0 -3.4 µm (ECFP-F2-5K, AK01, Spherotech Inc.).  FSC and SSC were calibrated with Rosetta Calibration (Exometry B.V., v1.13, section S1.10).  Concentrations reported in the manuscript describe the number of particles that fulfil the gating criteria per mL. |
| 4.4.1. Gate description | To automatically apply gates, generate pdf reports with scatter plots, and summarize the data in a table, custom-build software (MATLAB R2020b, MathWorks) was used. Please find below a description of the gates. First, only events that were collected during time intervals, for which the count rate was within 25% of the median count rate, were included. Second, platelets were excluded by applying a gate at the side scattering cross section (<2,000 nm^2^) and, depending on the fluorescence label, at a fluorescence channel. Third, events with a diameter <1,000 nm as measured by SSC after light scatter calibration (section S1.10) were included. Fourth, events positive for either APC, FITC, or PE were included. Fifth, fluorescence gates were automatically determined with a mathematical algorithm (MATLAB R2020b, MathWorks) and applied.  We applied a lower gate to the scatter of 10.15 nm^2^ and fluorescence detectors (185 APC MESF, 400 FITC MESF and 123 PE MESF) that is equal to the lower detection limit at the least sensitive measurement day over the time course of 12 months. |
| 4.4.2. Gate statistics | The number of positive events was corrected for flow rate, measurement time and dilutions performed during sample preparation. |
| 4.4.3. Gate boundaries | Please see section 4.1 |

APC: allophycocyanin; CD: cluster of differentiation; EDTA: Ethylenediamine tetraacetic acid; FITC: fluorescein isothiocyanate; FSC: forward scattering; MESF: Molecules of Equivalent Soluble Fluorochrome: PE: phycoerythrin; PMT: photomultiplier tube; SSC: side scattering.
