# Supplementary material for: Plasma extracellular vesicle test sample to standardize flow cytometry measurements
Source: Res Pract Thromb Haemost. 2023 May 18;7(4):100181. doi: 10.1016/j.rpth.2023.100181 (PMC10394550; doi:10.1016/j.rpth.2023.100181)
Supplement: Supporting Information 2 [file mmc2.docx]

**Supporting Information 2: Material and Methods for Figure 2, *“*Stable, ready-to-use test sample containing stained extracellular vesicles from human plasma to standardize flow cytometry measurements*”***

- 1. **Experimental design**

The aim of the flow cytometry experiment was to show that the plasma extracellular vesicle (EV) test sample (PEVTES) resembles subcellular particles in human plasma. To generate Figure 4 immuno-fluorescently stained plasma and freeze-thawed PEVTES that were stored in dimethyl sulfoxide (DMSO), glycerol or trehalose for 6 months at -80 °C were measured with a flow cytometer (Apogee A60-Micro, Apogee Flow Systems) of which the flow rate, fluorescence signals and light scattering signals were calibrated. To compare subcellular particles in human plasma to already immuno-fluorescently stained EVs in PEVTES plasma was immuno-fluorescently stained with CD61-allophycocyanin (APC) and erythrocyte-derived (CD235a- phycoerythrin (PE), to measure the concentration of platelet-derived and erythrocyte-derived EVs. Please note that even though the plasma sample was double stained only CD61-APC was used for the analysis. A description of how the PEVTES was developed and immuno-fluorescently stained can be found in the manuscript.

- 1. **Blood collection and preparation of plasma**

Blood was collected from 1 healthy and overnight fasting individuals with informed consent who denied having a disease and/or using medication. Venous blood was collected using a 21-Gauge needle (368607, Becton Dickinson (BD) Biosciences), and the first 3.5 mL of blood was discarded. Three tubes of EDTA blood (6 mL, 9203871, BD Biosciences) were collected per donor, mixed gently with the anti-coagulant, and processed within 15 minutes.

To prepare plasma, whole blood was centrifuged at 2,500 g, 15 minutes, 20 °C, acceleration speed 9, deceleration speed 1 using a Rotina 380 R equipped with a swing-out rotor and radius of 155 mm (Hettich Zentrifugen). Plasma was collected 10 mm (determined with a Lego brick) above the buffy coat using a plastic Pasteur pipette (86.1171.001, SARSTEDT), and transferred into a new 15-mL polypropylene centrifuge tube (62.9924272, SARSTEDT) Subsequently, the plasma was centrifuged at the same settings used for whole blood. Afterwards, plasma was collected to 10 mm above the pellet to reduce platelet contamination, transferred into a new 15-mL polypropylene centrifuge tube (62.9924272, SARSTEDT). Next, plasma was pooled, mixed gently and transferred to 1.5-mL low protein binding Eppendorf tubes (616201, Greiner Bio-One B.V., Alphen aan den Rijn, The Netherlands).

- 1. **EV staining**

The plasma sample was 20-fold pre-diluted in Dulbecco's Buffered Saline (dPBS; 21-031-CVR, Corning) to event rates below 5,000/s to further prevent swarm [1]. To measure the concentration of platelet-derived (CD61-APC), and erythrocyte-derived (CD235a-PE) EVs, plasma EVs were immuno-fluorescently stained. Before staining, aggregates present in the antibodies were removed by centrifugation at 18,890 g for 5 minutes at 20 °C. The supernatant minus 10 μL of the starting volume was collected and used for staining. EVs were stained with anti-human CD61-APC antibody (17-0619-42, VI-PL2, final concentration (f.c.) 8.33 μg/mL; eBioscience), anti-human CD235a-PE antibody (R7078, JC159, f.c. 100 μg/mL; Dako).

Table S2.1 contains an overview of the staining reagents. 1000 μL pre-diluted plasma were incubated for 2 hours at room temperature in the dark, with 137.5 μL of each antibody. Afterward, 9000 μL dPBS were added and the stained plasma sample was measured by flow cytometry.

- 1. **Flow rate quantification**

On the measurement day, we used 110 nm FITC beads with a specified concentration

(Apogee calibration beads, Apogee Flow Systems) to calibrate the

flow rate of the A60-Micro. As the A60-Micro is equipped with a syringe pump with

volumetric control, we assumed a flow rate of 3.01 μL/min for all measurements.

- 1. **Fluorescence calibration**

Calibration of the fluorescence detectors from arbitrary units (a.u.) to molecules of equivalent

soluble fluorochrome (MESF) was accomplished using 2 μm Q-APC beads (2321-175, Becton Dickinson (BD) Biosciences), and SPHERO PE Calibration Particle Kit, 3.0 -3.4 μm (ECFP-F2-5K, AK01, Spherotech Inc.). Calibrations of the APC and PE detectors were performed on 12.03.2020. For each measurement, we added fluorescent intensities in MESF to the flow cytometry data files by custom-build software (MATLAB R2020b, MathWorks) using following equation:

| $\text{I(MESF)}={10}^{a\cdot\log_{10} \text{I(a.u.)}+b}$ | Equation S1 |
| --- | --- |

where I, is the fluorescence intensity, and *a* and *b* are the slope and the intercept of the linear fits respectively, see table S2.2.

- 1. **Light scattering calibration**

Rosetta Calibration (Exometry B.V.) was used to relate scatter measured by forward scattering (FSC) and side scattering (SSC) to the effective scattering cross section and diameter of EVs. We modelled EVs as core-shell particles with a core refractive index of 1.38, a shell refractive index of 1.48, and a shell thickness of 6 nm. For each measurement, we added the FSC and SSC cross sections and EV diameters to the flow cytometry datafiles by custom-build software (MATLAB R2020b, MathWorks).

- 1. **Flow cytometry**

The concentration of EVs in the stained plasma sample and PEVTES stored for 6 months at -80°C was measured with a calibrated flow cytometer (A60-Micro, Apogee Flow Systems) equipped with a 405 nm laser (100 mW), 488 nm laser (100 mW) and 638 nm laser (100 mW). Prior to measurement, samples were pre-diluted in dPBS to event rates below 5,000/s to further prevent swarm [2]. Samples were measured for 120 seconds at a flow rate of 3.0 µl/minute. The trigger detector was side scattering (SSC) operating at 405-nm illumination wavelength and the trigger threshold was set to 14 arbitrary units, corresponding to a side scattering cross section of 10 nm^2^ (Rosetta Calibration, Exometry B.V.). For forward scattering (FSC) and SSC, the voltages were 380 V and 350 V, respectively. For all detectors, the peak height was analysed. APC signals were collected with a 638-D Red (Peak) detector (long pass 652 nm filter, PMT voltage 510 V). PE signals were collected with the 488-Orange (Peak) detector (575/30 nm band pass filter, PMT voltage 520 V). The EV concentration in this experiment describes the number of particles that exceed the side scatter threshold, with a diameter <1000 nm as measured by SSC after light scattering calibration, and events positive on the relevant fluorescence detectors.

A lower gate was set on 170 APC MESF for both the stained plasma and the PEVTES sample.

- 1. **Software and statistics**

Data analysis was performed by custom-build software (MATLAB R2020b, MathWorks) to automate data calibration and data processing. Graphs were made with custom-build software (MATLAB R2020b, MathWorks) and Adobe Illustrator (V 26.2.1, Adobe Inc.)

- 1. **Data sharing**

<https://doi.org/10.6084/m9.figshare.22323382.v3>; <https://doi.org/10.6084/m9.figshare.22323385.v2>

- 1. **References**

1 van der POL E, van GEMERT MJC, STURK A, NIEUWLAND R, van LEEUWEN TG. Single vs. swarm detection of microparticles and exosomes by flow cytometry. *Journal of Thrombosis and Haemostasis* John Wiley & Sons, Ltd; 2012; **10**: 919–30.

2 van der POL E, van GEMERT MJC, STURK A, NIEUWLAND R, van LEEUWEN TG. Single vs. swarm detection of microparticles and exosomes by flow cytometry. *Journal of Thrombosis and Haemostasis TA - TT -* 2012; **10**: 919–30.

**Figures and Tables**

**Table S2.1: Overview of staining reagents for the staining of plasma.**  Characteristics being measured, analyte, analyte detector, reporter, isotype, clone, concentration, manufacturer, catalog number and lot number of used staining reagents.

| **Characteristic**  **measured** | **Analyte** | **Analyte detector** | **Reporter** | **Isotype** | **Clone** | **Concentration during staining (µg mL^-1^)** | **Manufacturer** | **Catalog number** | **Lot number** |
| --- | --- | --- | --- | --- | --- | --- | --- | --- | --- |
| Integrin | Human  CD61 | Anti-human CD61 antibody | APC | IgG1 | VI-PL2 | 8.33 | eBioscience | 17-0619-42 | 2062626 |
| Glycoprotein | Human CD235a | Anti-human CD235a-PE antibody | PE | IgG1 | JC159 | 100 | Dako | R7078 | 20067598 |

APC: allophycocyanin; CD: cluster of differentiation; IgG: Immunoglobulin G; PE: phycoerythrin.

**Table S2.2: Overview of fluorescence calibrations.**

| **Fluorophore** | **Calibration date** | **Slope** | **Intercept** | **R^2^** |
| --- | --- | --- | --- | --- |
| APC | 20.07.2020 | 1.21272818 | -2.024438911 | 0.9942 |
| PE | 20.07.2020 | 1.034021078 | -1.614190222 | 0.9993 |

APC: allophycocyanin; PE: phycoerythrin.
