# Supplementary material for: Plasma extracellular vesicle test sample to standardize flow cytometry measurements
Source: Res Pract Thromb Haemost. 2023 May 18;7(4):100181. doi: 10.1016/j.rpth.2023.100181 (PMC10394550; doi:10.1016/j.rpth.2023.100181)
Supplement: Supporting Information 3 [file mmc3.docx]

**Supporting Information 3: Material and Methods for Figure 4, *“*Stable, ready-to-use test sample containing stained extracellular vesicles from human plasma to standardize flow cytometry measurements*”***

- 1. **Experimental design**

The aim of the flow cytometry experiment was to investigate the bench stability of the PEVTES. With bench stability we mean the stability of the test sample after thawing when stored at room temperature.

To measure the concentration of platelet-derived EVs i) plasma was immuno-fluorescently stained with CD61-allophycocyanin (APC) and erythrocyte-derived (CD235a- phycoerythrin (PE). A fresh PEVTES, which was also immuno-fluorescently stained with CD61-APC and CD235a-PE was prepared fresh and either diluted in dimethyl sulfoxide (DMSO), or a combination of DMSO and Bovine Serum Albumin (BSA) to improve the samples bench stability. Samples were measured with a flow cytometer (Apogee A60-Micro, Apogee Flow Systems) of which the flow rate, fluorescence signals and light scattering signals were calibrated. Please note that even though the plasma sample and PEVTES were double stained only CD61-APC was used for the analysis.

**Blood collection and preparation of plasma**

Blood was collected from 1 healthy and overnight fasting individuals with informed consent who denied having a disease and/or using medication. Venous blood was collected using a 21-Gauge needle (368607, Becton Dickinson (BD) Biosciences), and the first 3.5 mL of blood was discarded. One tube of EDTA blood (6 mL, 9203871, BD Biosciences) was collected, mixed gently with the anti-coagulant, and processed within 15 minutes.

To prepare plasma, whole blood was centrifuged at 2,500 g, 15 minutes, 20 °C, acceleration speed 9, deceleration speed 1 using a Rotina 380 R equipped with a swing-out rotor and radius of 155 mm (Hettich Zentrifugen). Plasma was collected 10 mm (determined with a Lego brick) above the buffy coat using a plastic Pasteur pipette (86.1171.001, SARSTEDT), and transferred into a new 15-mL polypropylene centrifuge tube (62.9924272, SARSTEDT) Subsequently, the plasma was centrifuged at the same settings used for whole blood. Afterwards, plasma was collected to 10 mm above the pellet to reduce platelet contamination, transferred into a new 15-mL polypropylene centrifuge tube (62.9924272, SARSTEDT). Next, plasma was pooled, mixed gently and transferred to 1.5-mL low protein binding Eppendorf tubes (616201, Greiner Bio-One B.V.).

- 1. **EV staining of the plasma sample**

The plasma sample was 20-fold pre-diluted in Dulbecco's Buffered Saline (dPBS; 21-031-CVR) to event rates below 5,000/s to further prevent swarm [1]. To measure the concentration of platelet-derived (CD61-APC), and erythrocyte-derived (CD235a-PE) EVs, plasma EVs were immuno-fluorescently stained. Before staining, aggregates present in the antibodies were removed by centrifugation at 18,890 g for 5 minutes at 20 °C. The supernatant minus 10 μL of the starting volume was collected and used for staining. EVs were stained with anti-human CD61-APC antibody (17-0619-42, VI-PL2, final concentration (f.c.) 8.33 μg/mL; eBioscience), anti-human CD235a-PE antibody (R7078, JC159, f.c. 100 μg/mL; Dako).

Table S3.1 contains an overview of the staining reagents. 1000 μL pre-diluted plasma were incubated for 2 hours at room temperature in the dark, with 137.5 μL of each antibody. Afterward, 9000 μL dPBS were added and the sample was measured by flow cytometry.

- 1. **Preparation of the plasma EV test sample (PEVTES)**

Blood was collected as described in Section 1.2

- - 1. **EV staining of the plasma EV test sample (PEVTES)**

To measure the concentration of platelet-derived (CD61-APC), erythrocyte-derived (CD235a-PE) EVs, plasma EVs were immuno-fluorescently stained. Before staining, aggregates present in the antibodies and were removed by centrifugation at 18,890 g for 5 minutes at 20 °C. The supernatant minus 10 μL of the starting volume was collected and used for staining. EVs were stained with anti-human CD61-APC antibody (17-0619-42, VI-PL2, final concentration (f.c.) 25 μg/mL; eBioscience), anti-human CD235a-PE antibody (R7078, JC159, f.c. 100 μg/mL; Dako). Table S3.2 contains an overview of the staining reagents. 1000 μL pre-diluted plasma were incubated for 2 hours at room temperature in the dark, with 137.5 μL of each antibody. Afterward, 9000 μL dPBS were added and the sample was measured by flow cytometry.

- - 1. **Size-exclusion chromatography**

Next, to separate EVs from unbound dye, soluble proteins, and reduce lipoprotein particles, SEC was performed (qEVsingle/70 nm1004125; Izon Science). Therefore, 1 mL of the plasma containing the pre-stained EVs was loaded on each washed SEC column, followed by elution with dPBS (21-031-CVR, Corning). The first 3.5 mL eluate containing the void volume was discarded, after which the 1 mL fraction containing most EVs was collected and pooled.

- - 1. **Platelet removal with polycarbonate filters**

To remove remaining platelets from plasma, plasma was filtered using 0.8-µm pore-size polycarbonate membrane filter (ATTP02500, Isopore^TM^, Merck Millipore) with a diameter of 25 mm. Typically, this step reduces the residual platelet concentration 1.5·10^2^-fold [2].

- - 1. **Stabilization**

To improve the stability of the pre-labelled and SEC-isolated EVs, the cryopreservation agents, dimethyl sulfoxide (DMSO), glycerol and trehalose were selected based on literature and tested [3–8]. The optimal concentration of each cryopreservation agent for long-term stability was investigated in preliminary experiments (data not shown). The PEVTES was diluted 2x in either 20% DMSO (1.02931.500, f.c. 10%; Merck Millipore), To prepare the PEVTES + Bovine Serum Albumin (BSA) sample, additionally BSA (A9647, f.c. 0.5%, Sigma Aldrich) was added.

- 1. **Flow rate quantification**

On the measurement day, we used 110 nm FITC beads with a specified concentration

(Apogee calibration beads, Apogee Flow Systems) to calibrate the

flow rate of the A60-Micro. As the A60-Micro is equipped with a syringe pump with

volumetric control, we assumed a flow rate of 3.01 μL/min for all measurements.

- 1. **Fluorescence calibration**

Calibration of the fluorescence detectors from arbitrary units (a.u.) to molecules of equivalent

soluble fluorochrome (MESF) was accomplished using 2 μm Q-APC beads (2321-175, Becton Dickinson (BD) Biosciences), and SPHERO PE Calibration Particle Kit, 3.0 -3.4 μm (ECFP-F2-5K, AK01, Spherotech Inc.). Calibrations of the APC and PE detectors were performed on 20.07.20. For each measurement, we added fluorescent intensities in MESF to the flow cytometry data files by custom-build software (MATLAB R2020b, MathWorks) using following equation:

| $\text{I(MESF)}={10}^{a\cdot\log_{10} \text{I(a.u.)}+b}$ | Equation S1 |
| --- | --- |

where I, is the fluorescence intensity, and *a* and *b* are the slope and the intercept of the linear fits respectively, see Table S3.3.

- 1. **Light scattering calibration**

Rosetta Calibration (Exometry B.V.) was used to relate scatter measured by forward scattering (FSC) and side scattering (SSC) to the effective scattering cross section and diameter of EVs. We modelled EVs as core-shell particles with a core refractive index of 1.38, a shell refractive index of 1.48, and a shell thickness of 6 nm. For each measurement, we added the FSC and SSC cross sections and EV diameters to the flow cytometry datafiles by custom-build software (MATLAB R2020b, MathWorks).

- 1. **Flow cytometry**

The concentration of EVs in the stained plasma sample and PEVTES stored for 6 months at -80°C was measured with a calibrated flow cytometer (A60-Micro, Apogee Flow Systems) equipped with a 405 nm laser (100 mW), 488 nm laser (100 mW) and 638 nm laser (100 mW). Prior to measurement, samples were pre-diluted in dPBS to event rates below 5,000/s to further prevent swarm [9]. Plasma samples were pre-diluted 20x in dPBS, while the PEVTES were pre-diluted 10x in dPBS. Samples were measured for 120 seconds at a flow rate of 3.0 µl/minute. The trigger detector was side scattering (SSC) operating at 405-nm illumination wavelength and the trigger threshold was set to 14 arbitrary units, corresponding to a side scattering cross section of 10 nm^2^ (Rosetta Calibration, Exometry B.V.).

For forward scattering (FSC) and SSC, the voltages were 380 V and 350 V, respectively. For all detectors, the peak height was analysed. APC signals were collected with a 638-D Red (Peak) detector (long pass 652 nm filter, PMT voltage 510 V). PE signals were collected with the 488-Orange (Peak) detector (575/30 nm band pass filter, PMT voltage 520 V).

The EV concentration in this experiment describes the number of particles that exceed the side scatter threshold, with a diameter <1000 nm as measured by SSC after light scattering calibration, and events positive on the relevant fluorescence detectors.

For the plasma samples a lower gate was set on 185 APC MESF and 228 PE MESF. For the PEVTES and PEVTES + BSA sample the lower gate was set on 147 APC MESF and 105 PE MESF.

- 1. **Software and statistics**

Data analysis was performed by custom-build software (MATLAB R2020b, MathWorks) to automate data calibration and data processing. Graphs were made with custom-build software (MATLAB R2020b, MathWorks) and Adobe Illustrator (V 26.2.1, Adobe Inc).

- 1. **Data sharing**

Data is available via: <https://doi.org/10.6084/m9.figshare.22323445.v3>; https://doi.org/10.6084/m9.figshare.22323460.v2

- 1. **References**

1 van der POL E, van GEMERT MJC, STURK A, NIEUWLAND R, van LEEUWEN TG. Single vs. swarm detection of microparticles and exosomes by flow cytometry. *Journal of Thrombosis and Haemostasis* John Wiley & Sons, Ltd; 2012; **10**: 919–30.

2 Bettin B, Gasecka A, Li B, Dhondt B, Hendrix A, Nieuwland R, van der Pol E. Removal of platelets from blood plasma to improve the quality of extracellular vesicle research. *Journal of Thrombosis and Haemostasis* John Wiley & Sons, Ltd; 2022; **20**: 2679–85.

3 Jeyaram A, Jay SM. Preservation and Storage Stability of Extracellular Vesicles for Therapeutic Applications. *AAPS J* 2017; **20**: 1.

4 Bahr MM, Amer MS, Abo-El-Sooud K, Abdallah AN, El-Tookhy OS. Preservation techniques of stem cells extracellular vesicles: a gate for manufacturing of clinical grade therapeutic extracellular vesicles and long-term clinical trials. *Int J Vet Sci Med* Taylor & Francis; 2020; **8**: 1–8.

5 Sameti M, Bohr G, Ravi Kumar MN V, Kneuer C, Bakowsky U, Nacken M, Schmidt H, Lehr C-M. Stabilisation by freeze-drying of cationically modified silica nanoparticles for gene delivery. *Int J Pharm* 2003; **266**: 51–60.

6 Fonte P, Soares S, Costa A, Andrade JC, Seabra V, Reis S, Sarmento B. Effect of cryoprotectants on the porosity and stability of insulin-loaded PLGA nanoparticles after freeze-drying. *Biomatter* Landes Bioscience; 2012; **2**: 329–39.

7 Wu Y, Deng W, Klinke 2nd DJ. Exosomes: improved methods to characterize their morphology, RNA content, and surface protein biomarkers. *Analyst* 2015; **140**: 6631–42.

8 Bosch S, de Beaurepaire L, Allard M, Mosser M, Heichette C, Chrétien D, Jegou D, Bach J-M. Trehalose prevents aggregation of exosomes and cryodamage. *Sci Rep* 2016; **6**: 36162.

9 van der POL E, van GEMERT MJC, STURK A, NIEUWLAND R, van LEEUWEN TG. Single vs. swarm detection of microparticles and exosomes by flow cytometry. *Journal of Thrombosis and Haemostasis TA- TT -* 2012; **10**: 919–30.

**Figures and tables**

**Table S3.1: Overview of staining reagents for plasma.**  Characteristics being measured, analyte, analyte detector, reporter, isotype, clone, concentration, manufacturer, catalog number and lot number of used staining reagents.

| **Characteristic**  **measured** | **Analyte** | **Analyte detector** | **Reporter** | **Isotype** | **Clone** | **Concentration during staining (µg mL^-1^)** | **Manufacturer** | **Catalog number** | **Lot number** |
| --- | --- | --- | --- | --- | --- | --- | --- | --- | --- |
| Integrin | Human  CD61 | Anti-human CD61 antibody | APC | IgG1 | VI-PL2 | 8.33 | eBioscience | 17-0619-42 | 2062626 |
| Glycoprotein | Human CD235a | Anti-human CD235a-PE antibody | PE | IgG1 | JC159 | 100 | Dako | R7078 | 20079786 |

APC: allophycocyanin; CD: cluster of differentiation; IgG: Immunoglobulin G; PE: phycoerythrin.

**Table S3.2: Overview of staining reagents for plasma EV test sample (PEVTES).**  Characteristics being measured, analyte, analyte detector, reporter, isotype, clone, concentration, manufacturer, catalog number and lot number of used staining reagents.

| **Characteristic**  **measured** | **Analyte** | **Analyte detector** | **Reporter** | **Isotype** | **Clone** | **Concentration during staining (µg mL^-1^)** | **Manufacturer** | **Catalog number** | **Lot number** |
| --- | --- | --- | --- | --- | --- | --- | --- | --- | --- |
| Integrin | Human  CD61 | Anti-human CD61 antibody | APC | IgG1 | VI-PL2 | 8.33 | eBioscience | 17-0619-42 | 2062626 |
| Glycoprotein | Human CD235a | Anti-human CD235a-PE antibody | PE | IgG1 | JC159 | 100 | Dako | R7078 | 20067598 |

APC: allophycocyanin; CD: cluster of differentiation; IgG: Immunoglobulin G; PE: phycoerythrin.

**Table S3.3: Overview of fluorescence calibrations for plasma and the plasma EV test sample (PEVTES).**

| **Fluorophore** | **Calibration date** | **Slope** | **Intercept** | **R^2^** |
| --- | --- | --- | --- | --- |
| APC | 20.07.2020 | 1.21272818 | -2.024438911 | 0.9942 |
| PE | 20.07.2020 | 1.034021078 | -1.614190222 | 0.9993 |

APC: allophycocyanin; PE: phycoerythrin.

**Figure S3.1. Bench stability of the plasma EV test sample (PEVTES) compared to human plasma.**

We found that the bench stability of the developed plasma EV test sample (PEVTES) decreased with measurement time. Therefore, we developed a procedure to stabilize the developed PEVTES by supplementing it with additional protein such as Bovine Serum Albumin (BSA), called stable PEVTES. Samples were measured with a calibrated flow cytometer (Apogee A60-Micro, Apogee Flow Systems). Data is presented as EV concentration (mL^-1^) versus measurement time (minutes). Data (symbols) have been fitted with a linear function (lines), resulting in a slope of 3766291 and a x-intercept of -1677 for plasma, a slope of -332980 and x-intercept of 671.5 for the PEVTES, and a slope of 77709 and a x-intercept of -2983 for the PEVTES + BSA. To prepare the PEVTES + BSA sample (stable PEVTES), BSA (f.c. 0.5%, Sigma Aldrich) has been added.
