# Supplementary material for: Plasma extracellular vesicle test sample to standardize flow cytometry measurements
Source: Res Pract Thromb Haemost. 2023 May 18;7(4):100181. doi: 10.1016/j.rpth.2023.100181 (PMC10394550; doi:10.1016/j.rpth.2023.100181)
Supplement: Supporting Information 4 [file mmc4.docx]

**Supporting Information 4: *“*Stable, ready-to-use test sample containing stained extracellular vesicles from human plasma to standardize flow cytometry measurements*”***

- 1. **Experimental design**

The aim of the experiment was to investigate the hypothesis that the increase in human plasma and the PEVTES observed by flow cytometry (Figure 4) was due to evaporation. Therefore, we performed an experiment in which we pipetted 200 µL of pre-diluted pool plasma in each well of a 96-well plate.

- 1. **Blood collection and preparation of plasma**

Blood was collected from 1 healthy and overnight fasting individuals with informed consent who denied having a disease and/or using medication. Venous blood was collected using a 21-Gauge needle (368607, Becton Dickinson (BD) Biosciences), and the first 3.5 mL of blood was discarded. One tube of EDTA blood (6 mL, 9203871, BD Biosciences) was collected, mixed gently with the anti-coagulant, and processed within 15 minutes.

To prepare plasma, whole blood was centrifuged at 2,500 g, 15 minutes, 20 °C, acceleration speed 9, deceleration speed 1 using a Rotina 380 R equipped with a swing-out rotor and radius of 155 mm (Hettich Zentrifugen). Plasma was collected 10 mm (determined with a Lego brick) above the buffy coat using a plastic Pasteur pipette (86.1171.001, SARSTEDT), and transferred into a new 15-mL polypropylene centrifuge tube (62.9924272, SARSTEDT) Subsequently, the plasma was centrifuged at the same settings used for whole blood. Afterwards, plasma was collected to 10 mm above the pellet to reduce platelet contamination, transferred into a new 15-mL polypropylene centrifuge tube (62.9924272, SARSTEDT). Next, plasma was pooled, mixed gently and transferred to 1.5-mL low protein binding Eppendorf tubes (616201, Greiner Bio-One B.V.). The new plasma sample is called pooled plasma.

- 1. **Determination of evaporation**

We determined the evaporation based on weight every hour over a period of 7 hours. Therefore, we pipetted 200 µL of 100x diluted pool plasma in each well of a 96-well plate (655101, Greiner Bio-One B.V.). The plate was kept at room temperature for 7 hours and weight twice every hour with a BP 3100 P scale (Sartorius). The average decrease per hour was calculated and plotted.

- 1. **Data sharing**

Data is available via: <https://doi.org/10.6084/m9.figshare.22347703.v2>

**Figures and tables**

**Figure S4.1.** To investigate our hypothesis that the increase in particle concentration in human plasma and our PEVTES sample was due to evaporation we performed an experiment in which we pipetted 200 µL of pre-diluted pool plasma in each well of a 96-well plate. We determined the evaporation based on weight every hour over a period of 7 hours. Data is presented as weight (kg) versus measurement time (hours). Each data point presents the mean of two measurements. Data (symbols) have been fitted with a linear function (lines), resulting in a slope of -0.0004855 and a x-intercept of -39.65.
